# Global visualization of adolescent idiopathic scoliosis treatment: a bibliometric analysis

**DOI:** 10.3389/fped.2025.1526459

**Published:** 2025-02-04

**Authors:** Xiaodong Zheng, Wenjin Han, Shan Gao, MengLong Jia, LiJun Liu, Longtan Yu

**Affiliations:** ^1^ The First Clinical School, Shandong University of Traditional Chinese Medicine, Jinan, China; ^2^ Department of Orthopedic Spine, Weifang Hospital of Traditional Chinese Medicine, Weifang, China; ^3^ School of Nursing, Xi’an Jiaotong University Health Science Center, Xi’an, China

**Keywords:** bibliometrics, adolescent idiopathic scoliosis, treatment, CiteSpace, visual analysis bibliometrics, visual analysis

## Abstract

**Objective:**

The objective of this study is to present a comprehensive overview of current advancements, prominent areas of interest, emerging trends, and frontiers in global research concerning the treatment of adolescent idiopathic scoliosis. The aim is to offer valuable insights and guidance for future research endeavors in this field.

**Methods:**

We conducted a literature search on the treatment of adolescent idiopathic scoliosis using the Web of Science Core Collection database spanning from 2009 to 2024. Visualization of countries, institutions, authors, and keywords was performed using CiteSpace.

**Results:**

1,002 English articles were included in this study. Between 2009 and 2020, publications remained relatively low and stable. However, in 2021, interest in the treatment of adolescent idiopathic scoliosis began to surge, a noticeable increase in publications, with a peak reached in 2022. The United States emerged as the leading contributor among countries, institutions, and authors. Nanjing University and Peter O. Newton were the most prolific institutions and authors, respectively. The academic journal with the highest number of published articles was Spine. Notably, areas such as Cobb angle measurement, the efficacy of non-surgical treatments for AIS, and factors influencing the success of brace treatment garnered significant attention as hot topics in adolescent idiopathic scoliosis treatment.

**Conclusion:**

The prognosis remains consistent across patients with varying degrees of curvature in adolescent idiopathic scoliosis. Given the limited understanding of its etiology, the relationship between curvature and prognosis, as well as the exploration of its underlying causes, could emerge as new research directions in the future.

## 1 Introduction

Adolescent idiopathic scoliosis is the primary cause of three-dimensional spine deformities. It is officially defined by the Scoliosis Research Society as a spinal deformity characterized by a coronal Cobb angle exceeding 10° (1). Adolescent idiopathic scoliosis is an abnormal curvature of the spine that occurs in late childhood or adolescence (2, 3). Adolescence is a pivotal period marked by self-perception and self-esteem development, a time when developmental changes such as scoliosis can profoundly impact both the physical and mental health of young individuals. This stage of life is particularly sensitive to psychological development, with research indicating that adolescents with scoliosis are more susceptible than adults to experiencing psychological distress. Such distress can significantly diminish their quality of life (4). Therefore, it is necessary to conduct in-depth research on the treatment of adolescent idiopathic scoliosis.

Adolescent Idiopathic Scoliosis (AIS) is a common spinal deformity in adolescents, with treatment options varying based on individual differences. Current methods primarily include observation, bracing, physical therapy, and surgical intervention. Observation is suitable for patients with mild curvature, and when combined with sensors, it allows for real-time monitoring. Bracing treatments utilize innovative technologies like 3D printing and dynamic braces to enhance comfort and effectiveness (5). Physical therapy can incorporate virtual reality to assist with posture correction. Surgical treatments focus on minimally invasive techniques and navigational applications, improving both safety and correction outcomes (6). Additionally, the application of biomaterials and robotic technologies offers emerging treatment options. By integrating genetic analysis and intelligent algorithms, precise predictions of disease progression and personalized treatment plans can be achieved. The application of new technologies is advancing the scientific and individualized approach to AIS treatment.

While numerous scholars have explored adolescent idiopathic scoliosis, the field lacks comprehensive visualization analysis, hindering the identification of current research trends and future directions. Visual research methodologies can illuminate rigorous data, uncover research hotspots and frontiers, and offer detailed insights into the dynamic development and overall structure of the field. This paper seeks to bridge this gap by employing bibliometric and visual analysis to summarize the current landscape, highlight hotspots, identify emerging trends, and delineate frontiers in global research on adolescent idiopathic scoliosis. The findings aim to provide theoretical foundations for future investigations in related areas.

## 2 Methods

### 2.1 Literature search queries

While databases like PubMed, Elsevier, and others serve the purpose of literature research, Web of Science stands out as the premier choice for bibliometric analysis. Therefore, the literature included in this study is sourced exclusively from the Web of Science Core Collection (WOSCC). The search query employed is as follows: (Adolescent* OR Adolescence OR Teen* OR Teenager* OR Youth*) AND (“idiopathic scoliosis”) AND (Therapeutic OR Therapy OR Therapie* OR Treatment*).

### 2.2 Literature screening

A systematic literature search was conducted on the treatment of adolescent idiopathic scoliosis from 2009 to 2024, focusing on articles in the English language. A total of 3,961 articles were initially identified. Inclusion criteria were set to include articles with essential information such as abstracts, keywords, and author affiliations, while exclusion criteria targeted literature unrelated to adolescent scoliosis treatment and non-article formats like conference abstracts, news reports, and letters. Following these criteria, 2,760 articles were screened, resulting in 1,201 articles deemed highly relevant. These 1,201 articles underwent detailed analysis to extract information regarding countries, institutions, authors, and keywords. It's important to note that this entire research process was conducted by a single researcher.

### 2.3 Analytical tools

In this literature analysis, we focused on examining countries, institutions, authors, keywords, and article citations. The visual representation comprises nodes and links, where nodes represent various entities such as countries, institutions, authors, keywords, and citations. The size of a node corresponds to the number of published papers associated with it, while the number and thickness of links between nodes indicate their proximity. Node and link colors signify different years. Additionally, the centrality of a node, indicating its importance and centrality in the knowledge network, is represented by the thickness of the purple ring surrounding it. A thicker purple ring denotes higher centrality. We configured CiteSpace with the following parameters: (1) a time span from January 2009 to April 2024, with each slice year equal to 1; (2) source of terms includes title, abstract, author's keyword, and keyword plus; (3) node types consist of country, institution, author, keyword, and reference; (4) threshold selection criteria were set to the first 50 items of each time slice, with other settings remaining at default values. For modular *Q*, a value greater than 0.3 indicates a significant cluster structure. Silhouette (S) values above 0.5 suggest reasonable clustering results, while values exceeding 0.7 indicate extremely reliable clustering outcomes.

## 3 Results

### 3.1 Literature search and screening results

In this study, a comprehensive search yielded a total of 3,961 English-language literature items. Through meticulous screening, 2,760 articles deemed irrelevant to the study's focus were excluded. Ultimately, 1,201 articles, meeting stringent inclusion and screening criteria, were identified as highly relevant and retained for further analysis.

### 3.2 Publication years and journals

**Figure 1:**
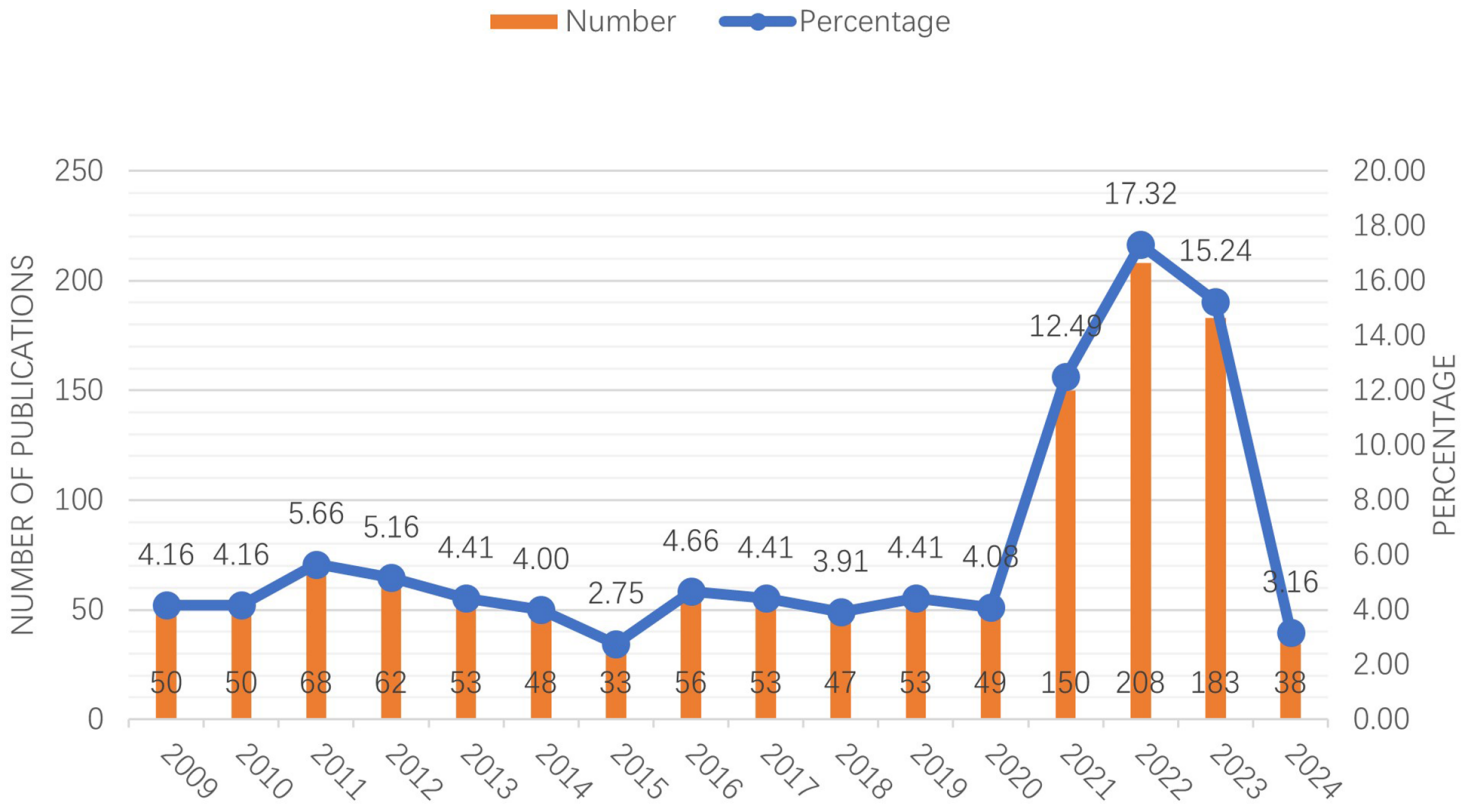
This figure illustrates the annual distribution of publications related to the treatment of adolescent idiopathic scoliosis from 2009 to 2024. The blue line represents the proportion of papers published each year relative to the total, while the orange bars indicate the absolute number of papers published annually. The data shows a steady increase in publications starting in 2021, peaking in 2022, and maintaining high levels in subsequent years, reflecting a growing interest and focus on this topic within the academic community.

A total of 1,201 articles on the treatment of adolescent idiopathic scoliosis were retrieved from the Web of Science Core Collection (WOSCC) database, and all 1,201 were original research articles, accounting for 100% of the retrieved literature. Figure 1 depicts the annual distribution of these publications from 2009 to 2024. The blue line represents the proportion of articles published in a given year relative to the total, while the orange bar chart indicates the absolute number of papers published in each year. As illustrated in Figure 1, the number of publications remained relatively low and stable between 2009 and 2020. However, starting in 2021, there was a noticeable increase in the popularity of research on adolescent idiopathic scoliosis treatment, with the number of publications trending upward. This trend peaked in 2022, and its popularity has remained high over the past three years. This suggests a growing interest and emphasis on research in this area, potentially driven by advancements in treatment methods or an increased awareness of the condition.

The table (Table 1) highlights the distribution of articles across 269 journals from 2009 to 2024, focusing on adolescent idiopathic scoliosis treatment. Notably, Spine emerged as the leading publisher with 176 papers (14.65%), followed by the European Spine Journal with 129 papers (10.74%).

**Table 1:** Top 10 journals with the most publications on the treatment of adolescent idiopathic scoliosis.

Rank	Journal	Number (%)	Country	IF^a^	JCR quartile
1	Spine	176 (14.65)	USA	3	Q2
2	European Spine Journal	129 (10.74)	USA	2.8	Q2
3	Spine Deformity	67 (5.58)	GERMANY (FED REP GER)	1.6	Q3
4	Journal of Pediatric Orthopedics	50 (4.16)	USA	1.7	Q2
5	Spine Journal	31 (2.58)	USA	4.5	Q1
6	Journal of Clinical Medicine	30 (2.50)	SWITZERLAND	3.9	Q1
7	Children-Basel	24 (2.00)	SWITZERLAND	2.4	Q2
8	Bmc Musculoskeletal Disorders	24 (2.00)	ENGLAND	2.3	Q4
9	Journal of Bone and Joint Surgery-American Volume	23 (1.92)	USA	5.3	Q1
10	Clinical Spine Surgery	21 (1.75)	USA	1.9	Q3

^a^ The IF were obtained from the 2022 Journal Citation Reports (JCR).

The top 10 journals collectively accounted for 47.88% of all publications, with impact factors ranging between 1.7 and 5.3. Approximately 30% of these journals were positioned in the Q1 region, denoting high-quality publications. Remarkably, the Journal of Bone and Joint Surgery American Volume boasted the highest impact factor at 5.3, with other journals averaging an impact factor of 2.70.

### 3.3 Collaboration analysis

#### 3.3.1 Country-by-country cooperation analysis

**Figure 2:**
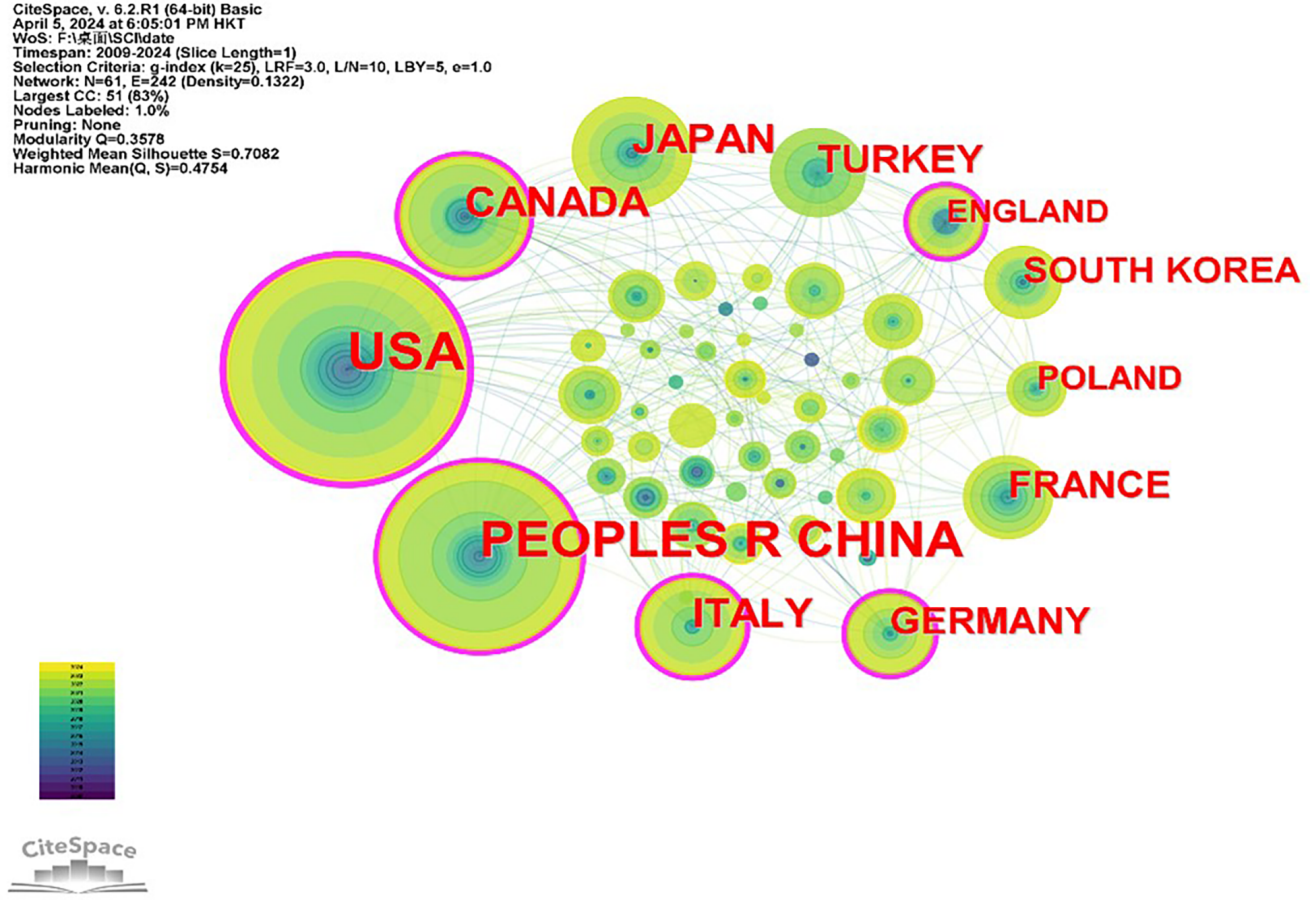
2013–2023 country cooperation network diagram.

The study of adolescent idiopathic scoliosis treatment demonstrates significant international collaboration, as depicted in Figure 2, a country/region network diagram comprising 61 nodes and 242 lines, with a network density of 0.1322. Leading the research efforts, the United States contributed the most, with 296 articles (22.46%), followed by China and Canada, with 209 (15.86%) and 89 articles (6.75%), respectively. Collectively, these top three countries accounted for 594 papers, representing 45.07% of the total. Regarding collaboration, the United States emerges as a central player with a centrality share of 0.36, followed by China at 0.18. Notably, the United States maintains robust collaboration with other countries, facilitating knowledge exchange and collective advancement in adolescent idiopathic scoliosis treatment research. (Refer to Table 2 for further details).

**Table 2:** Top 10 countries, institutions, and authors in the study.

No.	Countries/Regions	Count (%)	Centrality	Institution	Count (%)	Centrality	Authors	Count (%)	Centrality
1	USA	296 (22.46)	0.36	Nanjing University	22 (1.88)	0.00	Newton, Peter O	31 (2.27)	0.12
2	PEOPLES R CHINA	209 (15.86)	0.18	Shriners Hospitals Children Philadelphia	16 (1.37)	0.00	Qiu, Yong	26 (1.90)	0.04
3	CANADA	89 (6.75)	0.17	Univ Hong Kong	16 (1.37)	0.00	Shah, Suken A	25 (1.83)	0.03
4	JAPAN	85 (6.45)	0.03	Rady Childrens Hosp	15 (1.28)	0.00	Parent, Stefan	21 (1.54)	0.03
5	ITALY	68 (5.16)	0.11	Universite de Montreal	14 (1.20)	0.00	Zhu, Zezhang	18 (1.32)	0.00
6	TURKEY	58 (4.40)	0.06	University of Hong Kong	13 (1.11)	0.00	Samdani, Amer F	17 (1.24)	0.01
7	FRANCE	47 (3.57)	0.01	UDICE-French Research Universities	13 (1.11)	0.00	Yaszay, Burt	16 (1.17)	0.01
8	GERMANY	47 (3.57)	0.16	Childrens Hosp Philadelphia	13 (1.11)	0.00	Bastrom, Tracey P	15 (1.10)	0.01
9	SOUTH KOREA	41 (3.11)	0.00	Rady Childrens Hospital San Diego	13 (1.11)	0.00	Sudo, Hideki	13 (0.95)	0.04
10	POLAND	30 (2.28)	0.07	Chinese University of Hong Kong	13 (1.11)	0.00	Cheung, Jason Pui Yin	12 (0.88)	0.05

#### 3.3.2 Analysis of inter-agency collaboration

**Figure 3:**
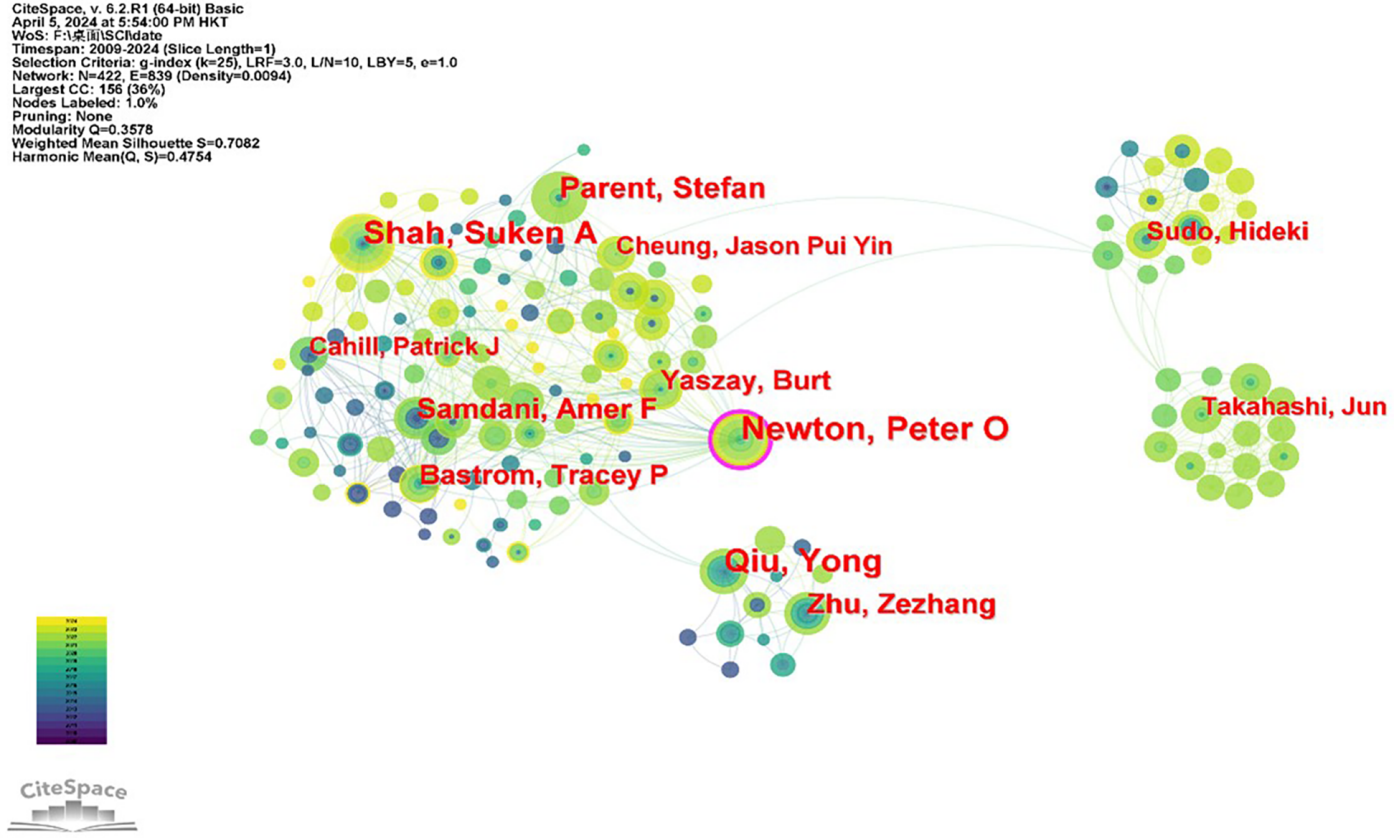
Diagram of the collaboration network between the institutions of the study from 2009 to 2024.

This paper presents a network diagram, depicted in Figure 3, which illustrates the collaborative relationships between institutions involved in research on adolescent idiopathic scoliosis treatment. The network comprises 359 nodes representing different institutions and 847 links indicating collaborative ties between them, with a network density of 0.0132. Notably, Nanjing University emerges as a significant contributor, with 22 papers (1.88%), followed by Shriners Hospitals for Children in Philadelphia with 16 papers (1.37%). Universities dominate the list of top contributors, representing 60% of the top 10 institutions based on the number of papers published. However, despite the prolific output of these institutions, the analysis reveals relatively weak cooperation among them, as indicated by the correlation of statistical agencies. This suggests opportunities for enhancing collaboration to foster greater synergy and impact in the field of adolescent idiopathic scoliosis treatment research. (Refer to Table 2 for further details).

#### 3.3.3 A study of the correlation between authors

**Figure 4:**
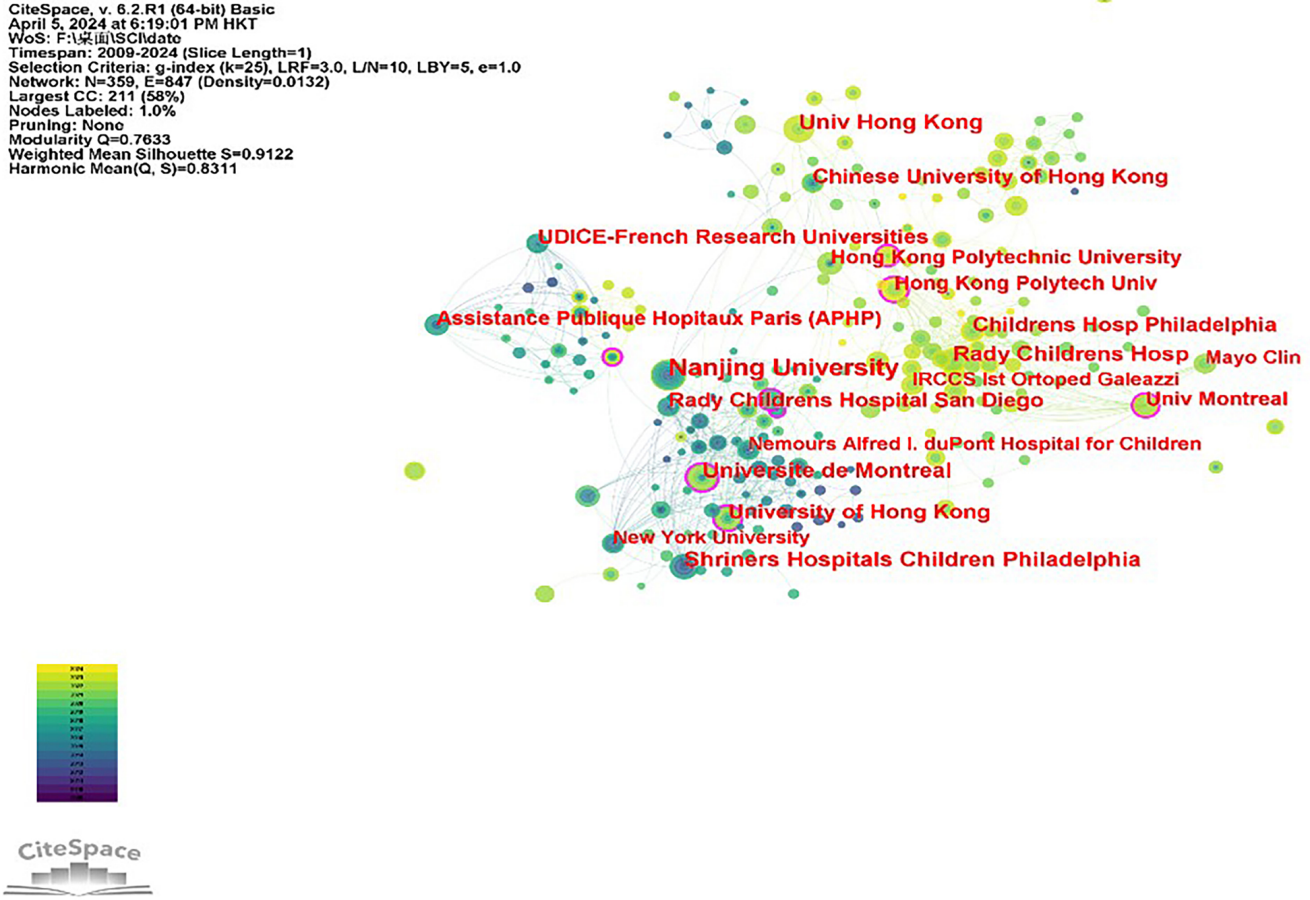
Network diagram of collaboration between authors and authors of the study, 2009–2024.

The research delves into the core authors contributing to the literature on adolescent idiopathic scoliosis treatment, analyzing their collaborative relationships through a network diagram (Figure 4). This diagram comprises 422 nodes representing authors and 839 links indicating collaboration between them, with a network density of 0.0094. Despite the low network density, signaling limited collaboration among authors, discernible node and team relationships emerge within the network diagram. Leading the pack, Newton and Peter O stand out with 31 papers (2.27%), followed closely by Qiu and Yong with 26 papers (1.90%), and Shah and Suken A with 25 papers (1.83%). The data presented in Table 2 underscore the relatively low relevance among authors, suggesting a lack of robust collaboration within the authorship community. This observation underscores potential avenues for enhancing cooperation and knowledge exchange among authors in the field of adolescent idiopathic scoliosis treatment research.

### 3.4 Keyword clustering

**Figure 5:**
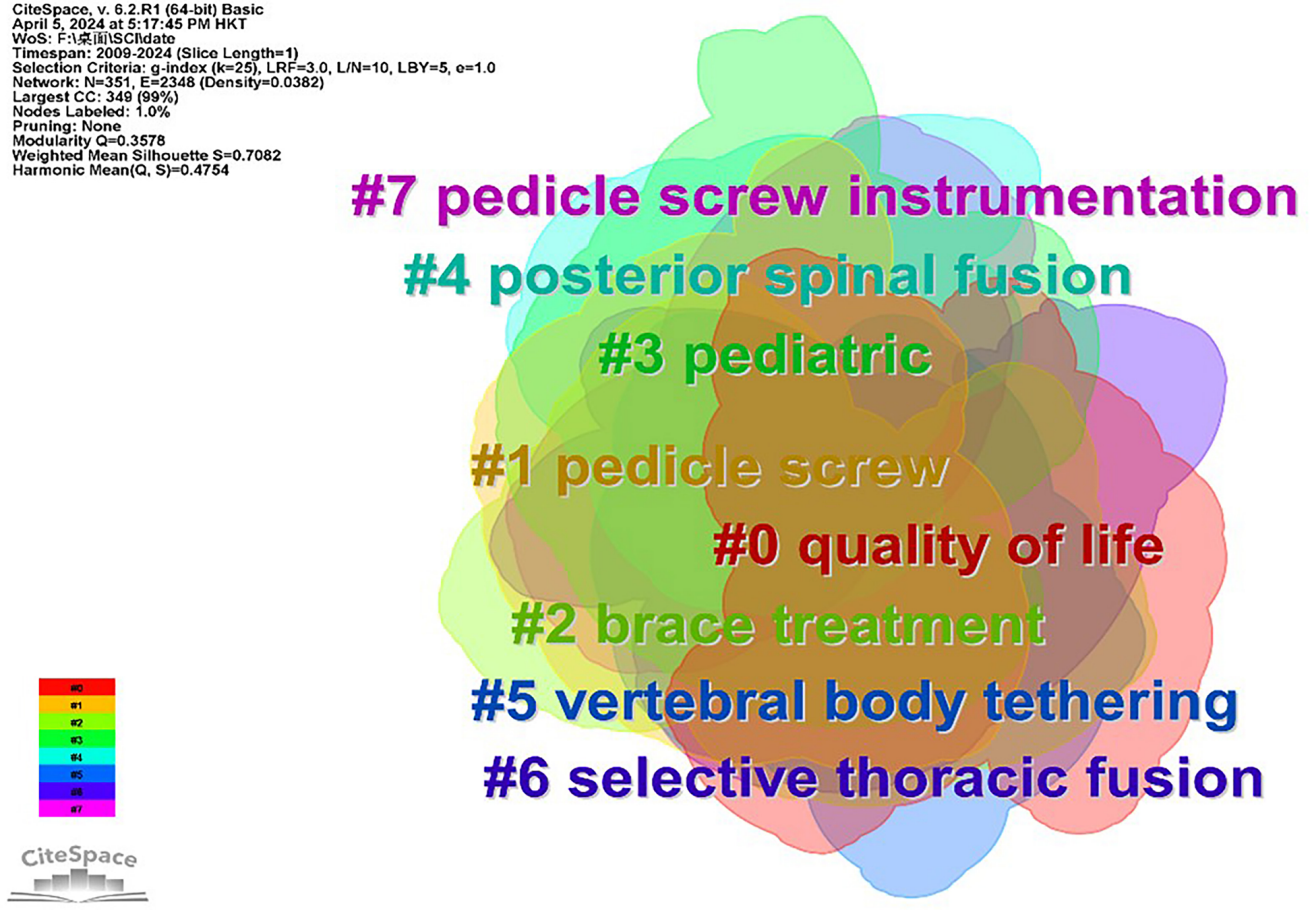
Keyword clustering analysis network diagram of the study from 2009 to 2024.

Keyword clustering offers insights into hotspots and trends in the treatment of adolescent idiopathic scoliosis. The analysis encompasses 351 nodes and 2,348 links, with a network density of 0.0382. Key terms prevalent in adolescent idiopathic scoliosis treatment include “adolescent idiopathic scoliosis,” “surgery,” and “instrumentation,” among others (refer to Table 3). Noteworthy centrality measures include “children,” “follow up,” “surgical treatment,” “fusion,” and “outcome” (refer to Table 3). Through clustering, the keywords coalesce into eight distinct groups, elucidating various facets of treatment approaches. These clusters encompass “quality of life,” “pedicle screw,” “brace treatment,” “pediatric,” “posterior spinal fusion,” “vertebral body tethering,” “selective thoracic fusion,” and “pedicle screw instrumentation” (refer to Figure 5). The rationality of this clustering is substantiated by *Q* = 0.3578 (>0.3) and *S* = 0.7082 (>0.5), affirming the coherence and relevance of the identified clusters in delineating trends and focal areas in adolescent idiopathic scoliosis treatment research.

**Table 3:** Keyword centrality table.

No.	Keywords	Counts	Keywords	Centrality
1	adolescent idiopathic scoliosis	354	children	0.12
2	surgery	90	follow up	0.12
3	instrumentation	88	surgical treatment	0.11
4	quality of life	79	fusion	0.10
5	surgical treatment	76	outcome	0.10
6	fusion	73	spinal deformity	0.09
7	idiopathic scoliosis	62	posterior spinal fusion	0.08
8	posterior spinal fusion	56	deformity	0.08
9	pedicle screw	51	reliability	0.08
10	outcome	51	brace	0.08

### 3.5 Keyword prominence analysis

**Figure 6:**
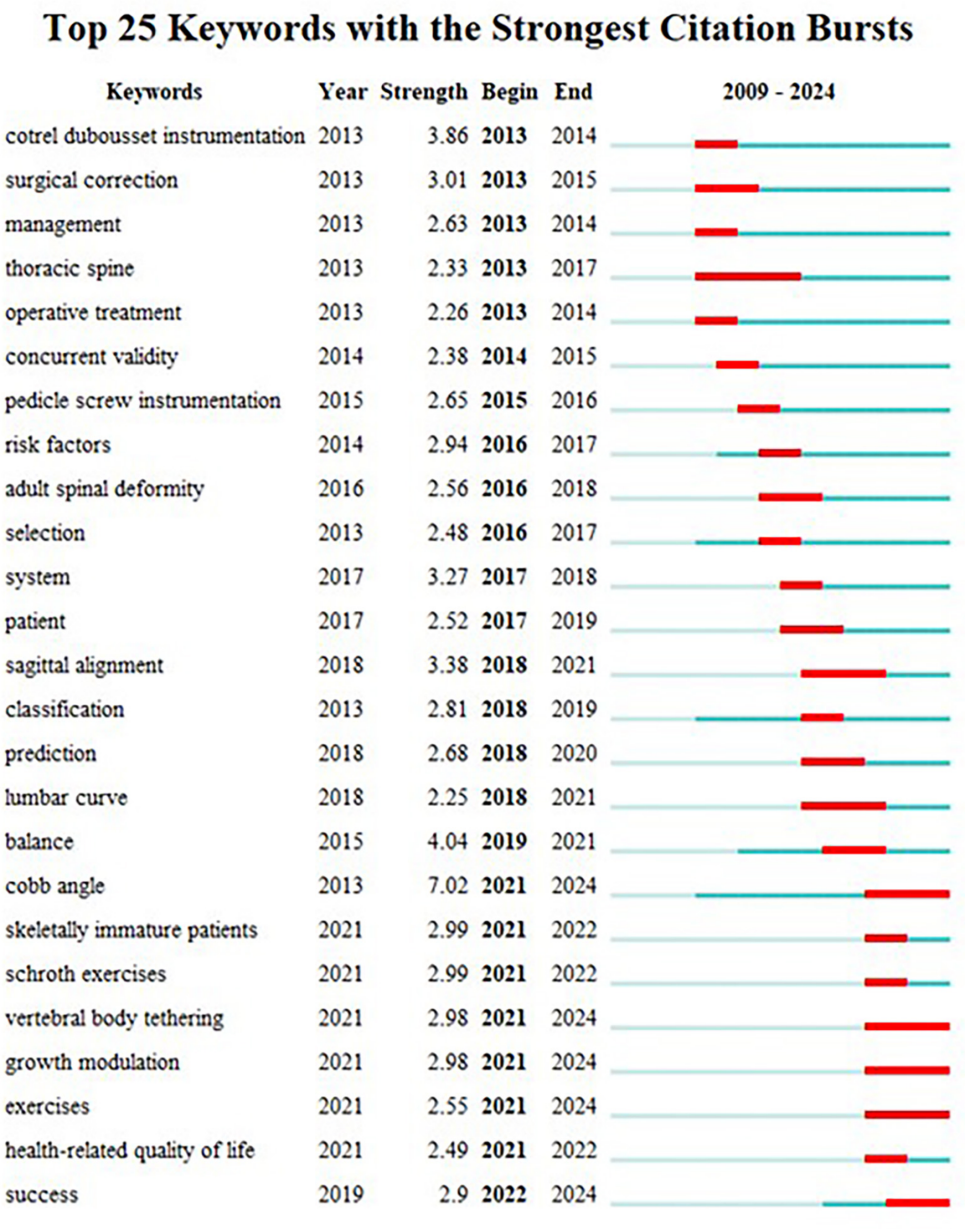
Keyword prominence network diagram from 2013 to 2023.

The utilization of keyword highlighting serves the purpose of scrutinizing the frontier direction of adolescent scoliosis treatment. Notably, the visualization of keywords in CiteSpace reveals a significant surge in their numbers within a condensed timeframe, underscoring the intensity and temporal dynamics of keyword outbreaks (refer to Figure 6). The investigation identifies 25 keywords that persisted for over a year in the study of adolescent scoliosis treatment, exhibiting a mean intensity exceeding 2. Leading the pack, “Cobb Angle” emerges with the highest burst intensity of 7.02. Notably, the keyword “thoracic spine” maintained prominence over the period 2013–2017. Furthermore, keywords such as “skeletally immature patients,” “Schroth exercises,” “vertebral body tethering,” “growth modulation,” “exercises,” “health-related quality of life,” and “success” have sustained outbreaks, extending into the present and signaling potential as future research hotspots. These findings offer valuable insights into emerging trends and focal areas within the realm of adolescent scoliosis treatment research.

### 3.6 Co-citation timeline analysis

**Figure 7:**
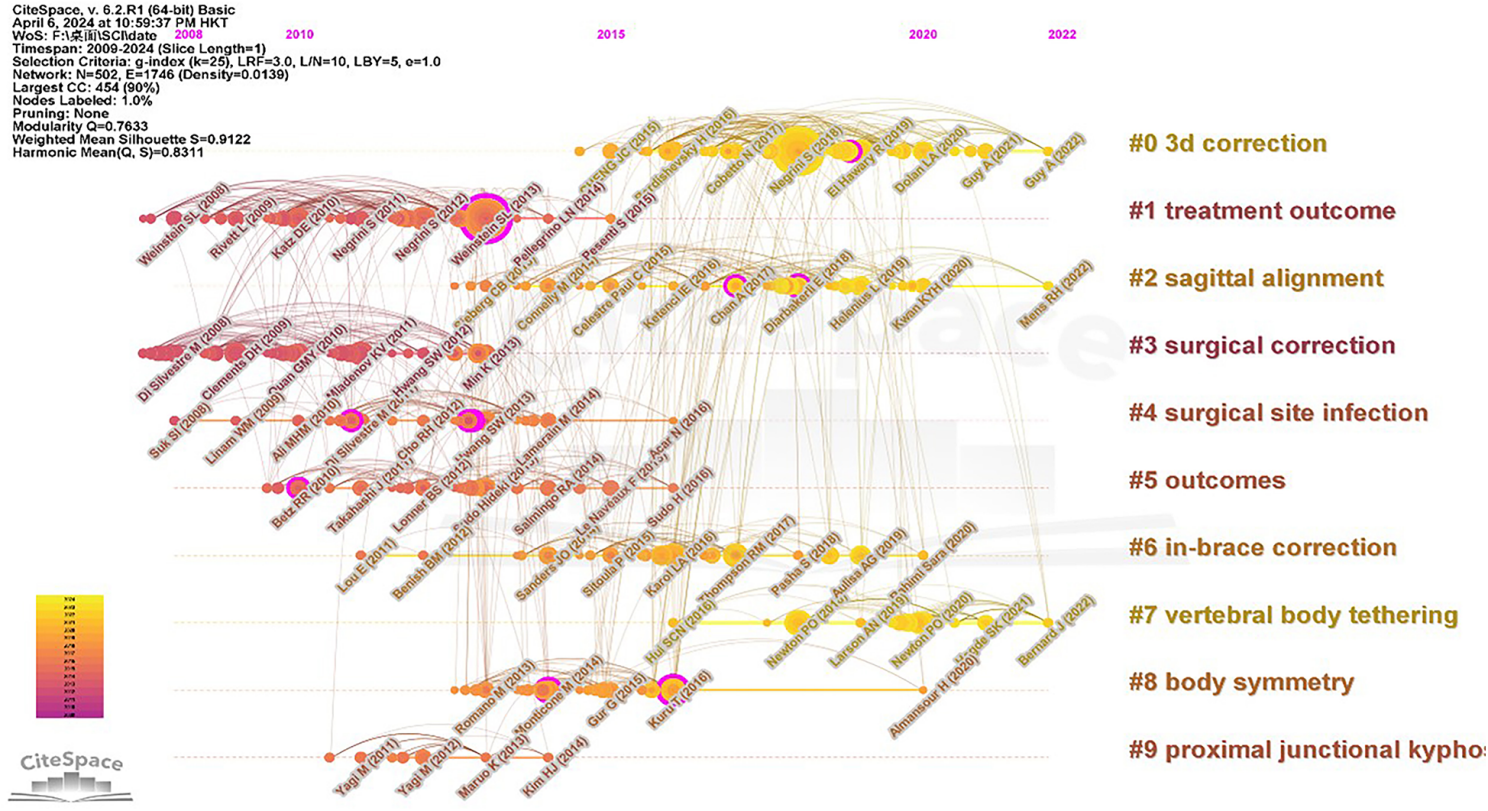
Co-citation timeline network from 2009 to 2024.

By scrutinizing the co-citation timeline, we glean insights into the evolution and trends shaping adolescent scoliosis treatment (refer to Figure 7). The network diagram encompasses 502 nodes and 1,746 network links, yielding a network density of 0.0139. Notably, the diagram is delineated into 10 distinct sets, with “3D Correction” emerging as the largest set, followed by “Treatment Outcome” and “Sagittal Alignment.”

The analysis reveals a high modularity index (*Q* = 0.7633) and a substantial silhouette score (*S* = 0.9122), affirming the rationality of the clustering approach. Moreover, there's a discernible increase in the number of nodes post-2012, with a concentration primarily observed between 2012 and 2016. Among the sets, notable citation burst nodes include “3D orthopedics” in group 0, “treatment outcomes” in group 1, and “surgical orthopedics” in group 3, underscoring pivotal areas of focus and innovation within adolescent scoliosis treatment research. These findings elucidate the trajectory of advancements and highlight promising avenues for further exploration in the field.

This article highlights 10 seminal works in the domain of adolescent scoliosis treatment (Table 4). Notably, these publications span the years 2010–2020, with approximately 80% being featured in Q1 journals. Impressively, 80% of the selected literature boasts an Impact Factor (IF) exceeding 3. The top-ranked papers include a clinical guideline authored by Stefano Negrini et al., offering comprehensive insights into idiopathic scoliosis treatment. Additionally, Weinstein, SL et al.'s publication delves into the pivotal role of braces in managing adolescent idiopathic scoliosis. Among the top 10 articles, two stand out as the most cited, garnering a total of 44 citations, while also boasting the highest Impact Factor (IF = 158.5). The remaining eight articles comprise a mix of research studies and clinical guidelines, collectively contributing to the evolving landscape of adolescent scoliosis treatment research.

**Table 4:** Representative articles in adolescent scoliosis treatment.

No.	Count	Centrality	Title	Author	Journal (JCR ®)	IF^a^	Published year
1	44	0.1	2016 SOSORT guidelines: orthopaedic and rehabilitation treatment of idiopathic scoliosis during growth	Stefano Negrini	N/A^b^	N/A^b^	2018
2	33	0.49	Effects of Bracing in Adolescents with Idiopathic Scoliosis	Weinstein, SL	MEDICINE, GENERAL & INTERNAL (Q1)	158.5	2013
3	13	0.01	Anterior Spinal Growth Tethering for Skeletally Immature Patients with Scoliosis A Retrospective Look Two to Four Years Postoperatively	Newton, PO	ORTHOPEDICS in SCIE edition (Q1)	5.3	2018
4	12	0.03	Effect of Compliance Counseling on Brace Use and Success in Patients with Adolescent Idiopathic Scoliosis	Karol, LA	ORTHOPEDICS in SCIE edition (Q1)	5.3	2016
5	12	0.24	The efficacy of three-dimensional Schroth exercises in adolescent idiopathic scoliosis: a randomised controlled clinical trial	Kuru, T	REHABILITATION in SCIE edition (Q1)	3	2016
6	12	0.02	2011 SOSORT guidelines: Orthopedic and Rehabilitation treatment of idiopathic scoliosis during growth	Negrini S	N/A^b^	N/A^b^	2012
7	12	0.01	Brace treatment in adolescent idiopathic scoliosis: risk factors for failure-a literature review	El Hawary, R	ORTHOPEDICS in SCIE edition (Q1)	4.5	2019
8	11	0.03	Brace Success Is Related to Curve Type in Patients with Adolescent Idiopathic Scoliosis	Thompson, RM	ORTHOPEDICS in SCIE edition (Q1)	5.3	2017
9	10	0.02	Anterior Spinal Growth Modulation in Skeletally Immature Patients with Idiopathic Scoliosis A Comparison with Posterior Spinal Fusion at 2 to 5 Years Postoperatively	Newton, PO	ORTHOPEDICS in SCIE edition (Q1)	5.3	2020
10	9	0.08	Brace Wear Control of Curve Progression in Adolescent Idiopathic Scoliosis	Katz, DE	ORTHOPEDICS in SCIE edition (Q1)	5.3	2010

^a^ The IF were obtained from the 2022 Journal Citation Reports (JCR).

^b^ Not applicable.

## 4 Discussion

This article employs bibliometric analysis to elucidate the landscape of adolescent idiopathic scoliosis treatment, shedding light on its current status, focal areas, and emerging frontiers. Over the decade spanning from 2009 to 2019, the volume of publications in this domain remained relatively stable, with a discernible uptick observed from 2020 onwards, culminating in a peak in 2022.

These scholarly contributions predominantly find their place in journals specializing in scoliosis research, with Spine emerging as the primary outlet for dissemination. Notably, the United States and China, alongside various academic institutions, stand out as the leading contributors, showcasing both the highest publication output and significant influence in the realm of adolescent idiopathic scoliosis research literature. This comprehensive analysis provides valuable insights into the trajectory and geographical distribution of research efforts in this critical area of healthcare.

Newton, Peter O emerges as the most prolific author in this field, having published the largest number of papers. However, while there are some network links between authors, the overall collaboration is relatively limited and dispersed. This suggests a low level of collaboration among authors, albeit with scattered connections, indicative of a lack of consistent and stable partnerships. The current research hotspots in adolescent idiopathic scoliosis (AIS) focus on the relationship between the quality of life of AIS patients and various treatments, including Schroth exercises, vertebral body tethering, and growth modulation. There is also an emerging interest in fusion-related treatments, suggesting a possible new trend in future studies.

The United States leads in this field, holding the most central position with the highest number of publications. It is followed by China and Canada, with China and Turkey being the only developing countries among the top 10 in terms of publication count. This highlights the prominence of developed countries in AIS research, with their more robust healthcare infrastructure and research funding. At the same time, the presence of developing countries among the top contributors indicates a growing global interest in scoliosis treatment research. Developed countries, such as the United States, have better economic conditions than developing countries, and their residents enjoy higher access to insurance plans and better economic standards (7). In developed countries, patients with conditions like scoliosis may have greater access to healthcare services, including specialized treatments and participation in research studies. This increased access can lead to more active involvement in scoliosis research and publication activities compared to patients in developing countries, where healthcare resources and research opportunities may be more limited.

Therefore, we recommend that scholars from China and Turkey, especially the former, conduct more collaborative research with other developed countries. Most of the top 10 institutions are from the United States, and 60% are universities. The network map of collaboration between institutions and authors is relatively fragmented. A growing body of evidence suggests that more inter-agency communication and collaboration between authors may be associated with higher research productivity and research quality (8, 9). Therefore, it is necessary to expand the network of cooperation between institutions and authors, especially with American universities.

Keyword clustering is a valuable technique for summarizing the core and hot spots within a research field. In the realm of AIS treatment, three prominent areas emerge as hot spots: Cobb angle measurement, the efficacy of non-surgical treatments, and the influencing factors affecting the success of brace treatment. At present, there are three main research directions in this field: interventions, influencing factors, and prognostic outcomes, but there are few studies on how to choose treatment methods based on different regions and different ethnic groups.

Notably, the Cobb angle is the most popular topic. The Cobb angle plays an important role in the diagnosis and treatment of adolescent idiopathic scoliosis and is not diagnosed until the Cobb angle exceeds 10° on standing spinal x-rays (10). Severe curvature of the Cobb angle >50° should be surgical (11). Patients with immature bones with a Cobb angle of ≥25° should use a brace to prevent or delay scoliosis progression. There is prognostic uncertainty about the Cobb angle of <25° and the growth of remaining bone, and doctors use a watchful waiting strategy (12). At present, there are three main types of intervention for Cobb angle: surgical treatment, brace treatment, and conservative treatment. In the actual treatment process, the best treatment plan should be selected according to the actual situation of the patient. Other treatments, such as enriched bone marrow obtained by selective cell preservation technology, have a positive effect on the treatment of adolescent idiopathic scoliosis and the improvement of the Cobb angle. However, the intensity, duration, and duration of treatment need to be further verified. Researchers should conduct high-quality randomized controlled trials of different treatment regimens to provide the most effective treatment to improve the Cobb angle (7–13). Other treatments, such as enriched bone marrow obtained by selective cell preservation technology, have a positive effect on the treatment of adolescent idiopathic scoliosis and the improvement of Cobb angle (14), but the intensity, duration, and duration of treatment need to be further validated. Researchers should conduct high-quality randomized controlled trials of different treatment options to provide the most effective treatment to improve the Cobb angle.

Another hot topic is the Efficacy of non-surgical treatment of AIS. However, due to the high risk and high cost of surgical treatment, non-surgical treatment has gradually become the primary choice, especially for patients with mild initial scoliosis, exercise therapy is a more effective and preventive method for scoliosis (15). At present, there are two types of exercise therapy, one is general exercise therapy, which mainly includes lower-intensity stretching exercises and strength training, which can stabilize the condition and alleviate the condition to a certain extent (16). The other approach is Physiotherapy Scoliosis Specific Exercise (PSSE), which focuses on preventing the development of scoliosis and is recommended by the SOSORT Association as the first step in the treatment of mild AIS (17). Through many high-quality studies, it has been found that PSSE is superior to general exercise therapy in correcting Cobb angle in AIS patients, but PSSE also has its disadvantages, especially in improving deformity, its efficacy is not as good as that of brace therapy, but compared with brace treatment, PSSE has obvious advantages in relieving pain and improving patients' mental health (18).

The third hotspot is the factors affecting the success of brace treatment, AIS is a relatively common disease, and there are two main treatment methods: surgical treatment and non-surgical treatment. Brace therapy has been shown to be one of the most effective non-surgical treatments (19). However, the success of brace therapy is affected by a variety of factors, among which adherence is the most important factor, and maintaining a high degree of adherence is the basis for the success of brace therapy (20). Increasing patient brace compliance during treatment can improve treatment outcomes (21). The length of time the brace is worn is also an important factor. It has been reported that factors associated with bracing may interfere with various aspects of a patient's health, negatively impacting the patient's daily life (22). Therefore, wearing a brace for a longer period each day may lead to a further decline in the quality of life of patients with AIS, which in turn affects patient compliance and thus disease progression (23). In addition to this, the main factors include the patient's low skeletal maturity and the initial Cobb angle >30° (24).

The emergence of keywords and the trend of their changes can reflect the development history and frontier of research. The process of this study can be divided into three processes. This study summarizes two research trends. The relationship between the first curvature and prognosis. To date, the effectiveness of school-age screening remains controversial in relevant studies, and it is uncertain what curvature can progress after diagnosis until the stage where treatment is needed (25). In response to this, research on the prognosis of adolescent idiopathic scoliosis has attracted increasing attention to guide the initiation of treatment (26). For brace therapy in particular, different curvatures have an important impact on the success of brace therapy (27). Future studies need to be carefully designed to explore the relationship between different curvatures and prognosis. Second, research on the etiology of adolescent idiopathic scoliosis. Current theories regarding the etiology of adolescent idiopathic scoliosis include genetic, metabolic, biomechanical, and environmental theories (28). At present, the pathogenesis of adolescent idiopathic scoliosis is mainly biomechanical, but there is a lack of accepted mechanistic theories (4, 25, 26, 28–30). In terms of genetic factors, although genetic factors are currently thought to play an important role in adolescent idiopathic scoliosis, this factor has considerable specificity (31, 32). Current research into the etiology of adolescent idiopathic scoliosis is complicated by the suspicion that it is not caused by a single cause, but by the interaction of multiple causes (4, 30–34). For adolescent idiopathic scoliosis, a better understanding of the underlying etiological mechanisms associated with it can lead to a better diagnosis of the disease and accurate prediction of deformity progression, helping patients achieve the best clinical outcomes (35).

This study carried out a comprehensive analysis focusing on various aspects such as the volume of annual publications, journal contributions, contributing countries, institutions, and authors, as well as keyword clustering and prominence. Through this analysis, we have charted the trends in annual publication volumes over the past fifteen years and identified the journals with the highest number of contributions. The findings suggest that enhanced collaboration among different countries, institutions, and authors is crucial. By examining keyword clustering and prominence, we have pinpointed current research hotspots and anticipated future research directions.

This study acknowledges certain limitations. Firstly, the search was confined to publications from the WoSCC database, excluding literature from other significant databases such as PubMed or Embase relevant to adolescent idiopathic scoliosis treatment. While each database has unique characteristics, combining papers from multiple sources can offer a more thorough understanding of the topic. While the WoSCC database is highly respected and contains prestigious academic journals, it's essential to consider literature from additional sources to ensure a comprehensive review. Secondly, due to constraints with CiteSpace software, this study only encompasses English articles published within the last fifteen years, potentially missing out on valuable research published in other languages or before the specified timeframe. This limitation might impact the study's breadth and applicability. To mitigate these limitations, future research could expand the search to encompass multiple databases and languages. Additionally, employing alternative bibliometric analysis tools could enhance the comprehensiveness of the review of adolescent idiopathic scoliosis treatment literature.

## 5 Conclusion

The study you're referring to analyzed a total of 1,002 English-language articles focused on the treatment of AIS from 2009 to 2022. It observed that while publication volume remained low and stable until 2020, there was a noticeable increase starting in 2021, with the highest number of publications recorded in 2022. This trend suggests a growing interest and development in the field of AIS treatment during these years. The analysis highlighted that the United States led in terms of contributions, with significant input from both Nanjing University and Peter O. Newton, identified as the most prolific institution and author, respectively. The journal “Spine” was noted as the primary publication venue for research in this area. Research hotspots identified include the Cobb angle, the efficacy of non-surgical treatments for AIS, and factors influencing the success of brace treatment. These focus areas have been crucial in understanding and advancing treatment strategies for AIS. The paper also points out emerging trends in the research, particularly the relationships between spinal curvature and patient prognosis, as well as the underlying causes of AIS. Investigating these areas could lead to innovative treatment approaches and more tailored care plans, potentially improving outcomes for those with AIS. The study underscores the importance of translating these research findings into clinical practice and serves as a valuable resource for scholars aiming to further explore AIS treatment strategies. This kind of research is vital not only for expanding scientific understanding but also for enhancing practical applications that benefit patients directly.
